# Prevalence, treatment, control of type 2 diabetes and the risk factors among elderly people in Shenzhen: results from the urban Chinese population

**DOI:** 10.1186/s12889-020-09045-1

**Published:** 2020-06-26

**Authors:** Yuanying Sun, Wenqing Ni, Xueli Yuan, Hongshan Chi, Jian Xu

**Affiliations:** Department of Health Management, Shenzhen Center for Chronic Disease Control, No.2021, Buxin Rd, Shenzhen, Guangdong 518020 PR China

**Keywords:** Type 2 diabetes, Prevalence, Treatment, Control, Elderly population

## Abstract

**Background:**

Type 2 diabetes is regarded as one of the major public health problems worldwide. We aim to investigate the prevalence, treatment, and control rate in the Chinese urban population aged 65 years or older and also identified associated risk factors.

**Methods:**

One hundred twenty-four thousand seven participants aged 65 years old and older were recruited from January 2018 through December 2018 at local community health service centers in Shenzhen. Fasting plasma glucose, as well as other biochemical indicators, were measured by standard methods. The analysis of multivariate logistic regression was applied to assess associated risk factors of type 2 diabetes.

**Results:**

Approximately 22.5% of elderly urban Chinese residents had diabetes. Among people with diabetes, 54.8% received medical treatment. Only 34.4% of those who were treated had their glycemic controlled. The prevalence of T2D increased with increasing age before 80 years old, male, inadequate active physical activity, drinking, previous history of CVD, higher BMI, central obesity, and hypertension.

**Conclusions:**

Our findings suggested that attention should be paid to the prevention and control of T2D in Chinese urban elderly population. The health policy department should develop effective strategies aimed at improving health care management of T2D in elderly adults.

## Background

Type 2 diabetes (T2D) is regarded as one of the major public health problems worldwide [1]. China has the world’s largest diabetes epidemic, with a substantially increasing trend with the rapid socio-economic development and lifestyle change in past decades. The prevalence of diabetes and prediabetes in China was 10.9 and 35.7% in 2013, according to a national survey [2]. Studies verified that diabetes is associated with cardiovascular diseases and related death, which results in high medical expenditures [3, 4]. The International Diabetes Federation reported that 13% of health costs in China in 2010 was attributable to diabetes [5]. Older adults aged 65 years old and above are at higher risk of getting diabetes than the other generations. And according to reports, the prevalence of diabetes among the elderly is elevating recently [6, 7].

For the past decades, studies have proved well-established evidence that the progression of diabetes may be controlled through proper intervention [8–11]. Therefore, it is crucial to understand the current severity of diabetes in elderly adults.

Several previous regional surveys had been conducted to investigate the prevalence, treatment, and control of diabetes in China [12–16]. However, few of these studies were focused on the elderly population. Therefore, our study aimed to investigate the prevalence, treatment, and control rate of T2D among elderly population in urban China and also identified related risk factors to provide evidence for disease prevention and help elevate the life quality of older diabetic patients. Strategies targeting diabetes in the elderly population should be proposed.

## Methods

### Subjects and design

We recruited people 65 years old and above from the lists of all residents registered at local community health service centers from January 2018 through December 2018 in Shenzhen by convenience sampling. One hundred forty-one thousand six hundred eighty-four people were recruited into the study, which accounted for 36.9% (141,684/383,700) of the resident population of elderly adults in Shenzhen based on data from the 2015 population census. A questionnaire, blood sample test, and physical examinations were provided during the study. We collected data from local community health service centers. After excluding individuals with incomplete data, 124,007 subjects (87.5%) completed the entire study. All participants signed the written informed consent before entering the study. If the participants were not able to write or read, we contacted their relatives to inform them and signed on their behalf. The study protocol was approved by the ethics committee of Shenzhen Center for Chronic Disease Control (SZCCC-201802).

### Questionnaire survey

After blood collection, all participants were interviewed face to face. A standardized questionnaire was administered by trained staff to obtain information on sociodemographic information (e.g., date of birth, gender, educational level, etc.), previous history (e.g., history of previous disease, operation history, history of trauma, etc.), family history (e.g., hypertension, diabetes, coronary heart disease, chronic obstructive pulmonary disease, malignant tumor, stroke, etc.), lifestyle (exercise frequency, smoking status, drinking habit, etc.), and medication use under the supervision of trained general practitioners and nurses (Additional File 1). Weight, height, waist circumference (WC), systolic blood pressure (SBP), and diastolic blood pressure (DBP) were measured with the use of standard methods.

We defined the educational level as illiterate, primary education, junior school education and above. And marital status was classified as married or cohabiting, widowed, divorced and single. The definition of the answer “yes” in physical exercise was participants who take exercise that causes sweating and shortness of breath for more than once a week [17]. Participants’ drinking habit (habitual drinker, non-habitual drinker and non-drinker) and smoking status (current smoker, ex-smoker and never-smoker) were categorized as described elsewhere [18, 19].

The research staff members had completed the training course about the entire study and received a written project operation manual, which included detailed instructions on the questionnaire surveys, physical examination index measurement, and blood sampling.

### Physical examination

After overnight fasting, anthropometric examinations were performed in the morning. Physical examination measurements were taken according to standard procedures [20]. Height and weight were measured with the participants wearing light clothing without shoes. WC was measured horizontally at the midpoint of the midaxillary line between the twelfth rib head and the superior anterior spine, at the end of a normal exhalation. Body mass index (BMI) was defined as the weight (kg) divided by squared height (m^2^). By using standard-certified sphygmomanometers, the participants’ right arm blood pressure at the heart level was measured three times. We take the average of the results in three measurements as the final blood pressure value. Participants were prohibited from drinking coffee, smoking, or exercising, within 30 min before the physical examination.

### Blood sample collection and biochemical analysis

With more than 8 h of overnight fasting, participants’ vein blood samples were taken. The clinical laboratory at each grade 2 hospital (community health service center is directly affiliated to the hospital) analyzed the samples. All participating laboratories had completed a standardization and competency program. Fasting venous blood was drawn from subjects to measure the fasting plasma glucose (FPG) level by automatic biochemistry analyzer. Glucose oxidase measurements were used to determine the FPG level.

### Definitions

Participants were regarded as T2D if one of the following three conditions was met: (1) previously diagnosed by professional doctors, (2) FPG ≥ 7.0 mmol/L, (3) 2-h plasma glucose level ≥ 11.1 mmol/L. [21] Treatment was defined as the rate of people with diabetes who had taken diabetic medication. Control was defined as the percentage of diabetic patients with FPG < 7.0 mmol/L.

Hypertension was defined as SBP ≧140 mmHg and/or DBP≧ 90 mmHg and/or self-reported treatment with antihypertensive medication within 2 weeks [22]. We defined overweight as a BMI between 24.0 to 27.9, and obesity as a BMI ≥ 28.0. The definition of central obesity was WC equal or more than 85 cm in females and 90 cm in males according to the Healthy Adult Weight Determination in China (WS/T 428–2013) [23].

### Statistical analysis

Characteristics of the study subjects were described as mean ± standard deviation for continuous variables and number (percentage) for classified variables. In our analysis, continuous variables in different groups were analyzed by *t*-test or variance analysis, and classified variables were analyzed by the chi-square test. To detect risk factors of older diabetic patients, multivariate logistic regression models were estimated. Model fittings were conducted using backward elimination with a threshold of 0.10 for variable inclusion in the model. Odds ratios were calculated with 95% CIs. Statistically significant was regarded as a level of two-sided *P* < 0.05. SAS version 9.4 software (SAS Institute, Cary, NC, USA) was used in the data cleaning and analyzing process.

## Results

### General characteristics of participants

The sociodemographic characteristics of elderly population in Shenzhen were shown in Table 1. One hundred twenty-four thousand seven participants were investigated in the study. The study population had an average age of 71.3 years old. There were 54,649 male subjects (44.1%) and 69,358 female subjects (55.9%). The socio-demographic characteristics of the study population were statistically different in the two gender subgroups. The BMI, SBP, WC, FBG were higher in females than that in the male group. While males were older, had higher DBP, and a lower rate of CVD history. Also, the education level, marital status, physical activity status, smoking habit, and drinking habit were all statistically different between both genders.

**Table 1:** Sociodemographic characteristics of elderly participants in Shenzhen

Characteristics	General(*n* = 124,007)	Female(*n* = 69,358)	Male(*n* = 54,649)	Statistics	*P*
Age (years)	71.3 ± 5.6	71.1 ± 5.6	71.5 ± 5.5	*t* = 10.5	< 0.001
BMI (Kg/m^2^)	23.8 ± 3.2	23.9 ± 3.3	23.8 ± 3.0	*t* = −3.9	< 0.001
SBP (mm Hg)	134.7 ± 17.7	135.4 ± 18.0	133.9 ± 17.2	*t* = −15.0	< 0.001
DBP (mm Hg)	77.2 ± 10.3	76.4 ± 10.2	78.4 ± 10.3	*t* = 36.1	< 0.001
WC (cm)	85.1 ± 8.8	84.0 ± 8.8	86.5 ± 8.7	*t* = 51.1	< 0.001
FBG (mmol/L)	6.0 ± 1.9	6.0 ± 2.0	5.9 ± 1.9	*t* = −5.3	< 0.001
Education level, n (%)				χ^2^ = 4180.4	< 0.001
Illiterate	10,054 (8.1)	7640 (11.0)	2414 (4.4)		
Primary education	44,096 (35.6)	27,877 (40.2)	16,219 (29.7)		
Junior school education and above	69,857 (56.3)	33,841 (48.8)	36,016 (65.9)		
Marital status, n (%)				χ^2^ = 1277.3	< 0.001
Married or cohabiting	119,314 (96.2)	65,614 (94.6)	53,700 (98.3)		
Widowed	3623 (2.9)	3001 (4.3)	622 (1.1)		
Divorced	565 (0.5)	470 (0.7)	95 (0.2)		
Single	505 (0.4)	273 (0.4)	232 (0.4)		
Physical activity, n (%)				χ^2^ = 296.5	< 0.001
Yes	95,338 (76.9)	52,054 (75.0)	43,284 (79.2)		
No	28,669 (23.1)	17,304 (25.0)	11,365 (20.8)		
Smoking status, n (%)				χ^2^ = 21,984.6	< 0.001
Current smoker	10,163 (8.2)	703 (1.0)	9460 (17.3)		
Ex-smoker	7662 (6.2)	180 (0.3)	7482 (13.7)		
Never-smoker	106,182 (85.6)	68,475 (98.7)	37,707 (69.0)		
Drinking habit, n (%)				χ^2^ = 13,070.3	< 0.001
Non-drinker	103,388 (83.4)	65,253 (94.1)	38,135 (69.8)		
Non-habitual drinker	12,737 (10.3)	2783 (4.0)	9954 (18.2)		
Habitual drinker	7882 (6.3)	1322 (1.9)	6560 (12.0)		
History of cardiovascular disease, n (%)				χ^2^ = 99.7	< 0.001
Yes	5192 (4.2)	2549 (3.7)	2643 (4.8)		
No	118,815 (95.8)	66,809 (96.3)	52,006 (95.2)		

Continuous variables in different groups were analyzed by *t*-test or variance analysis and categorical variables were analyzed by the chi-square test

### Prevalence, treatment and control rates in different subgroups

As shown in Table 2, 22.5% of elderly urban Chinese residents had diabetes. Among people with diabetes, 54.8% received medical treatment. Only 34.4% of those who were treated had their glycemic controlled. The prevalence and treatment rate for T2D increased with age and then decreased in participants more than 80 years old (25.5, 56.9%). While older people aged 80 years old or more had a higher control rate (37.5%). The prevalence and treatment rate had no difference in males and females. However, men have a significantly higher rate of control than women (35.2% vs 33.7%). Also, higher treatment rate was found in the group with higher education levels (57.5% vs 50.3%). Single older participants had lower prevalence and treatment rates when compared with participants who had companions. Higher prevalence, treatment, and control rates were found in people with regular physical activity. Ex-smokers had the highest prevalence, treatment and control rates in contrast to current smokers and non-smokers. Habitual drinkers had the highest treatment rate and the lowest diabetic prevalence. Participants with the previous history of CVD, obesity, central obesity, or hypertension had higher prevalence and control rates than those without.

**Table 2:** The prevalence, treatment, and control rate of T2D among adults aged 65 and older

Characteristics	N	Prevalence (%)	Treatment (%)	Control (%)
Total	124,007	22.5%	54.8%	34.4%
Age groups				
65–69 years old	61,096	20.7	54.2	33.9
70–74 years old	33,246	23.5	54.9	33.8
75–79 years old	16,853	25.5	56.9	34.7
80 years old or more	12,812	24.5***	54.2**	37.5***
Gender				
Males	54,649	22.2	54.8	35.2
Females	69,358	22.7	54.8	33.7*
Education level				
Illiterate	10,054	22.3	50.3	35.9
Primary education	44,096	22.1	51.4	31.5
Junior school or higher	69,857	22.8*	57.5***	35.9***
Marital status				
Married or cohabiting	119,314	22.4	54.7	34.3
Widowed	3623	24.8	57.0	36.7
Divorced	565	26.0	62.6	42.9
Single	505	21.2***	46.7*	33.6
Physical activity				
Yes	95,338	21.7	55.9	34.9
No	28,669	22.7***	51.1***	32.4***
Smoking status				
Current smoker	10,163	23.1	53.6	31.4
Ex-smoker	7662	22.6	58.4	35.6
Never-smoker	106,182	20.3***	54.6**	34.5**
Drinking habit				
Non-drinker	103,388	20.3	55.1	34.4
Non-habitual drinker	12,737	21.1	56.7	35.1
Habitual drinker	7882	22.8***	47.6***	33.0
History of CVD				
Yes	5192	32.9	56.6	43.3
No	118,815	22.0***	54.7	33.8***
BMI				
Low weight	4562	11.4	49.8	42.5
Normal weight	62,574	20.4	56.2	36.7
Overweight	45,397	24.8	54.3	32.7
Obesity	11,474	29.4***	51.8***	29.9***
Central obesity				
Yes	49,844	26.6	55.4	31.8
No	74,163	19.7***	54.3	36.7***
Hypertension				
Yes	69,207	27.8	56.1	37.1
No	54,800	15.7***	51.8***	28.3***

* = *P* < 0.05; ** = *P* < 0.01; *** = *P* < 0.001. Categorical variables were analyzed by chi-square test

### Risk factors analyses on the prevalence, treatment and glycaemic control of diabetes

By applying univariate and multivariate logistic regression analysis, risk factors on the prevalence, treatment, and control of diabetes were found. For the results see Table 3. Older age (aOR = 1.2, 95%CI: 1.2–1.3), drinking (aOR = 1.2, 95%CI: 1.1–1.2), history of CVD (aOR = 1.5, 95%CI: 1.4–1.6), high BMI (aOR = 1.2, 95%CI: 1.2–1.3), central obesity (aOR = 1.2, 95%CI: 1.2–1.3), and history of hypertension (aOR = 1.9, 95%CI: 1.8–1.9) were risk factors of the prevalence of diabetes while physical activity (aOR = 0.9, 95%CI: 0.9–1.0) was negatively related to the prevalence rate. For elders with T2D, a higher education level (aOR = 1.3, 95%CI: 1.2–1.5), physical activity (aOR = 1.2, 95%CI: 1.1–1.2), ex-smoker (aOR = 1.2, 95%CI: 1.1–1.3), central obesity (aOR = 1.2, 95%CI: 1.1–1.2), and hypertension (aOR = 1.2, 95%CI: 1.1–1.3) were positively related to the treatment rate of diabetes. In contrast, habitual drinker (aOR = 0.7, 95%CI: 0.6–0.8) and high BMI (aOR = 0.7, 95%CI: 0.7–0.8) were negatively related to the treatment rate. Physical activity (aOR = 1.1, 95%CI: 1.0–1.2), low BMI (aOR = 1.4, 95%CI: 1.1–1.6), history of CVD (aOR = 1.4, 95%CI: 1.3-1.6) and hypertension (aOR = 1.5, 95%CI: 1.5–1.6) were positively related to the glycemic control of diabetes, while primary education (aOR = 0.8, 95%CI: 0.8–0.9), current smoker (aOR = 0.9, 95%CI: 0.8–1.0), and central obesity (aOR = 0.9, 95%CI: 0.8–0.9) were negatively related to that.

**Table 3:** Risk factors analyses on the prevalence, treatment, and glycaemic control of T2D (OR, 95% CI)

	Prevalence		Treatment		Control	
	OR(95%CI)^a^	aOR(95%CI)^b^	OR(95%CI)^a^	aOR(95%CI)^b^	OR(95%CI)^a^	aOR(95%CI)^b^
**Age groups**						
65–69 years old	1.0	1.0	1.0	1.0	1.0	1.0
70–74 years old	1.2 (1.1,1.2)*	1.1 (1.1,1.2)*	1.0 (0.9,1.1)	–	1.0 (0.9,1.1)	–
75–79 years old	1.3 (1.3,1.4)*	1.2 (1.2,1.3)*	1.1 (1.0,1.2)*	–	1.0 (1.0,1.1)	–
80 years old or more	1.2 (1.2,1.3)*	1.1 (1.1,1.2)*	1.0 (0.9,1.1)	–	1.2 (1.1,1.3)*	–
**Gender**						
Males	1.0	1.0	1.0	1.0	1.0	1.0
Females	1.0 (1.0,1.1)	1.0 (0.9,1.0)*	1.0 (1.0,1.1)	–	0.9 (0.9,1.0)*	–
**Education level**						
Illiterate	1.0	1.0	1.0	1.0	1.0	1.0
Primary education	1.0 (0.9,1.0)	–	1.0 (1.0,1.1)	1.0 (1.0,1.2)	0.8 (0.7,0.9)*	0.8 (0.8,0.9)*
Junior school or higher	1.0 (1.0,1.1)	–	1.3 (1.2,1.5)*	1.3 (1.2,1.5)*	1.0 (0.9,1.1)	1.0 (0.9,1.1)
**Marital status**						
Married or cohabiting	1.0	1.0	1.0	1.0	1.0	1.0
Widowed	1.1 (1.1,1.2)*	–	1.1 (1.0,1.3)	–	1.1 (1.0,1.3)	–
Divorced	1.2 (1.0,1.5)*	–	1.4 (1.0,1.9)	–	1.4 (1.0,2.0)*	–
Single	0.9 (0.8,1.2)	–	0.7 (0.5,1.1)	–	1.0 (0.7,1.5)	–
**Physical activity**						
Yes	0.9 (0.9,1.0)*	0.9 (0.9,1.0)*	1.2 (1.1,1.3)*	1.2 (1.1,1.2)*	1.1 (1.0,1.2)*	1.1 (1.0,1.2)*
No	1.0	1.0	1.0	1.0	1.0	1.0
**Smoking status**						
Current smoker	1.1 (1.1,1.2)*	–	1.0 (0.9,1.0)	1.0 (0.9,1.1)	0.8 (0.8,1.0)*	0.9 (0.8,1.0)*
Ex-smoker	1.0 (1.0,1.1)	–	1.2 (1.1,1.3)*	1.2 (1.1,1.3)*	1.0 (0.9,1.2)	1.0 (0.9,1.1)
Never-smoker	1.0	1.0	1.0	1.0	1.0	1.0
**Drinking habit**						
Non-drinker	1.0	1.0	1.0	1.0	1.0	1.0
Non-habitual drinker	1.1 (1.1,1.2)*	1.1 (1.1,1.2)*	1.1 (1.0,1.2)	1.0 (0.9,1.1)	1.0 (1.0,1.1)	–
Habitual drinker	1.1 (1.1,1.2)*	1.2 (1.1,1.2)*	0.7 (0.7,0.8)*	0. 7 (0.6,0.8)*	0.9 (0.8,1.0)	–
**History of CVD**						
Yes	1.7 (1.6,1.8)*	1.5 (1.4,1.6)*	1.1 (1.0,1.2)	–	1.5 (1.4,1.7)*	1.4 (1.3,1.6)*
No	1.0	1.0	1.0	1.0	1.0	1.0
**BMI**						
Low weight	0.5 (0.5,0.6)*	0.6 (0.5,1.6)	0.8 (0.6,0.9)*	0.9 (0.7,1.0)	1.3 (1.1,1.5)*	1.4 (1.1,1.6)*
Normal weight	1.0	1.0	1.0	1.0	1.0	1.0
Overweight	1.3 (1.2,1.3)*	1.1 (1.1,1.2)*	0.9 (0.9,1.0)*	0.9 (0.8,0.9)*	0.8 (0.8,0.9)*	0.9 (0.8,0.9)*
Obesity	1.6 (1.5,1.7)*	1.2 (1.2,1.3)*	0.8 (0.7,0.9)*	0.7 (0.7,0.8)*	0.7 (0.7,0.8)*	0.8 (0.7,0.9)*
**Central obesity**						
Yes	1.5 (1.4,1.5)*	1.2 (1.2,1.3)*	1.0 (1.0,1.1)	1.2 (1.1,1.2)*	0.8 (0.8,0.9)*	0.9 (0.8,0.9)*
No	1.0	1.0	1.0	1.0	1.0	1.0
**Hypertension**						
Yes	2.1 (2.0,2.1)*	1.9 (1.8,1.9)*	1.2 (1.1,1.3)*	1.2 (1.1,1.3)*	1.5 (1.4,1.6)*	1.5 (1.5,1.6)*
No	1.0	1.0	1.0	1.0	1.0	1.0

* = *P* < 0.05; *OR* Odds ratio, *aOR* Adjusted odds ratio, *CI* Confidence interval; OR (95%CI)^a^: Univariate logistic analysis; aOR (95%CI)^b^: Multivariate logistic analysis. Model fittings were conducted using backward elimination with a threshold of 0.10 for variable inclusion in the model.; Variables that were not included in the final multivariate regression model were marked with “-”

## Discussion

This study investigated the prevalence, treatment, and control of diabetes in a large representative sample in Shenzhen, China. We found that 22.5% of elderly urban Chinese residents had diabetes. Furthermore, among people with diabetes, 54.8% received medical treatment. Only 34.4% of those who were treated had their glycemic controlled. All the figures alarm that T2D has become a severe public health burden in the Chinese elderly urban population.

According to a national survey conducted in 2013, involving 170,287 Chinese residents, from 31 provinces, showed that the prevalence of total diabetes was 20.2% in people aged 60 years or older [2]. In our study, the prevalence of elderly urban population was higher. Several possible reasons may cause the difference: first, our study population was generally older than those in national research (greater or equal than 65 years old vs. greater or equal than 60 years old). Second, our study was conducted more recently than the national survey (2018 vs. 2013); Third, urban residents have more access to medical resources and higher quality medical services, which led to a higher chance of being diagnosed at the early stage of diabetes incident. Also, a national survey including 46,239 participants showed that the prevalence of diabetes and levels of metabolic risk factors were higher in urban cities compared with rural areas [24]. A higher rate of T2D prevalence leads to a greater amount of costs for society. To reduce the burden of diabetes and its complications, it is important to take interventions to elevate the treatment and control of diabetes.

In 2011, the rates of treatment and control of diabetes in urban China were 51.3 and 33.8%, according to a survey with 20,242 residents aged 18–79 years old [15]. A cross-sectional survey conducted in Northeast China indicated that the treatment and control rates of diabetes mellitus were 52.9 and 44.2% in 2012 for participants aged 18 to 79 years old [12]. A similar study investigated in rural northern Chinese population showed values of 57.7 and 41.5% [13]. In developed counties such as the US, the treatment and control rates in elderly population were 50.9 and 50.4%, respectively [25]. In our results, the treatment and control rates were 54.8 and 34.4%. The lower control rate in our study may result from the remarkable changes in lifestyles over the past decades. For example, the percentage of meat consumption grew consistently, and consumption of coarse grain decreased a lot. Also, due to the development of industrialization and transportation, city residents tend to be less physically active during the day. These changes all lead to the occurrence of chronic disease, which may be one of the reasons for the high prevalence of diabetes. Interventions avoiding the process of diabetes should focus on the risk factors that are associated with prevalence, treatment, and control rates of diabetes.

In our study, older people with higher educational levels were more likely to be treated, equally likely to have diabetes and be controlled. Previous studies verified that health literacy concerning diabetes prevention and control among elderly individuals was low in China [26]. Educational level was proved to be positively associated with the level of diabetes health literacy [27]. People with higher educational level may have access to more knowledge about diabetes management and control and were more willing to receive medical treatment.

Previous epidemiologic studies and our study have shown that BMI, WC, CVD history and hypertension are risk factors for the prevalence of diabetes [24, 28, 29]. We have made a consistent conclusion compared with previous studies results in older adults. Lifestyles such as smoking and drinking were significant risk factors of diabetes. We found that smoking is associated with higher diabetes prevalence and lower control rate, while drinking is associated with higher diabetes prevalence and lower treatment rates.

Increased activity has been shown to be associated with reduced type 2 diabetes risk [30]. In our study, older people with regular physical activity is negatively associated with the prevalence of T2D and positively associated with treatment and control of T2D. Physical activity may improve metabolic flexibility in skeletal muscle and thereby contribute to the prevention of T2D [31].

The strength of our research is that we provide up-to-date estimates of prevalence, treatment, and control of T2D among elderly adults in the urban Chinese population. However, several limitations should be considered. First, our research enrolled the elderly population by convenience sampling, which cannot represent the whole elderly population in Shenzhen. Second, variables such as dietary habits and family history are not available in the data, which are considered as potential risk factors of diabetes.

## Conclusions

Our research suggested that the prevalence of T2D was high and still increasing in the urban Chinese population. Also, improvements in treatment and control rates are needed. Our research provides insight into the controllable risk factors affecting the Chinese adult population aged 65 years and above. As this population is still growing in China, more efforts to improve the overall management of T2D in urban areas should be given.

## Supplementary information

**Additional file 1.** Questionnaire for residents aged 65 years or older in Shenzhen.

## Data Availability

The data that support the findings of this study are available from Shenzhen Center for Chronic Disease Control but restrictions apply to the availability of these data, which were used under license for the current study, and so are not publicly available.

## Abbreviations

T2D: Type 2 diabetes
CVD: Cardiovascular disease
CI: Confidence interval
BMI: Body mass index
SBP: Systolic blood pressure
DBP: Diastolic blood pressure
WC: Waist circumference
FPG: Fasting plasma glucose
OR: Odds ratio
