# Supplementary material for: Prevalence, treatment, control of type 2 diabetes and the risk factors among elderly people in Shenzhen: results from the urban Chinese population
Source: BMC Public Health. 2020 Jun 26;20:998. doi: 10.1186/s12889-020-09045-1 (PMC7318517; doi:10.1186/s12889-020-09045-1)
Supplement: Supplementary file 1 — Additional file 1. Questionnaire for residents aged 65 years or older in Shenzhen. [file 12889_2020_9045_MOESM1_ESM.docx]

**Additional file 1. Questionnaire for residents aged 65 years or older in Shenzhen**

No: _______________

| **Section 1 Basic Personal Information** | | | |
| --- | --- | --- | --- |
| 1. Name: __________ 2. ID Number: 🞏🞏🞏🞏🞏🞏🞏🞏🞏🞏🞏🞏🞏🞏🞏🞏🞏🞏 3. Gender: 🞏Female 🞏Male 4. Date of birth: 🞏🞏🞏🞏-🞏🞏-🞏🞏 5. Phone Number: 🞏🞏🞏🞏🞏🞏🞏🞏🞏🞏🞏 6. Home Address: ______________________________ 7. Blood Type: 🞏A 🞏B 🞏O 🞏AB 🞏Unknown 8. Educational Level: 🞏Postgraduate 🞏Undergraduate 🞏Technical secondary school education 🞏Senior high school 🞏Junior high school 🞏Primary school 🞏Illiterate 🞏Unknown 9. Occupation: 🞏Civil servant 🞏Technician 🞏Merchant 🞏Farmer 🞏Worker 🞏Soldier 🞏Other classifications 🞏 Unknown 10. Marital Status: 🞏 Married or cohabiting 🞏Widowed 🞏 Divorced   🞏Single | | | |
| **Section 2 Previous History** | | | |
| 1. History of previous disease | | | |
| Type of disease | | Date of diagnosis | |
| 🞏Hypertension | | 🞏🞏🞏🞏-🞏🞏-🞏🞏 | |
| 🞏Diabetes | | 🞏🞏🞏🞏-🞏🞏-🞏🞏 | |
| 🞏Coronary heart disease | | 🞏🞏🞏🞏-🞏🞏-🞏🞏 | |
| 🞏COPD | | 🞏🞏🞏🞏-🞏🞏-🞏🞏 | |
| 🞏Malignant tumor | | 🞏🞏🞏🞏-🞏🞏-🞏🞏 | |
| 🞏Stroke | | 🞏🞏🞏🞏-🞏🞏-🞏🞏 | |
| 🞏Mental disorder | | 🞏🞏🞏🞏-🞏🞏-🞏🞏 | |
| 🞏Tuberculosis | | 🞏🞏🞏🞏-🞏🞏-🞏🞏 | |
| 🞏Hepatitis | | 🞏🞏🞏🞏-🞏🞏-🞏🞏 | |
| 🞏Occupational disease | | 🞏🞏🞏🞏-🞏🞏-🞏🞏 | |
| 🞏Other diseases: _______ | | 🞏🞏🞏🞏-🞏🞏-🞏🞏 | |
| 1. Operation history: 🞏No 🞏Yes, ________, 🞏🞏🞏🞏-🞏🞏-🞏🞏 | | | |
| 1. History of trauma: 🞏No 🞏Yes, ________, 🞏🞏🞏🞏-🞏🞏-🞏🞏 | | | |
| 1. History of blood transfusion: 🞏No 🞏Yes, _____, 🞏🞏🞏🞏-🞏🞏-🞏🞏 | | | |
| 1. Family history | | | |
| Family member | | History of previous disease | |
| Father | | 🞏Hypertension 🞏Diabetes  🞏Coronary heart disease  🞏COPD 🞏Malignant tumor  🞏Stroke 🞏Mental disorder  🞏Tuberculosis 🞏Hepatitis  🞏Other diseases: _______ | |
| Mother | | 🞏Hypertension 🞏Diabetes  🞏Coronary heart disease  🞏COPD 🞏Malignant tumor  🞏Stroke 🞏Mental disorder  🞏Tuberculosis 🞏Hepatitis  🞏Other diseases: _______ | |
| Brothers and sisters | | 🞏Hypertension 🞏Diabetes  🞏Coronary heart disease  🞏COPD 🞏Malignant tumor  🞏Stroke 🞏Mental disorder  🞏Tuberculosis 🞏Hepatitis  🞏Other diseases: _______ | |
| Children | | 🞏Hypertension 🞏Diabetes  🞏Coronary heart disease  🞏COPD 🞏Malignant tumor  🞏Stroke 🞏Mental disorder  🞏Tuberculosis 🞏Hepatitis  🞏Other diseases: _______ | |
| **Section 3 Lifestyle and Personal Habit** | | | |
| 1. Exercise frequency: 🞏Everyday 🞏More than once a week   🞏Once a week or less 🞏Never   1. Average time per exercise: _____minutes 2. How long have you been exercising? _____months 3. What kind of exercise do you usually do? __________ 4. Smoking status: 🞏Current smoker 🞏Ex-smoker 🞏Never-smoker 5. Daily smoking amount: ________ cigarettes 6. Age of first smoking: ________ years old 7. Age of quitting smoking: ________ years old 8. Drinking habit: 🞏Non-drinker 🞏Non-habitual drinker   🞏Habitual drinker   1. Daily drinking amount: ________ ml 2. Age of first drinking: ________ years old 3. Have you ever been drunk in the last year? 🞏Yes 🞏No 4. What kind of wine do you usually drink?   🞏Chinese Liquor 🞏Beer 🞏Red wine 🞏Yellow rice wine  🞏Other types of wine: __________  29. Medication use (Drug name, dosage, medication compliance) | | | |
| Drug name | Dosage | Frequency | Medication compliance |
|  |  |  |  |
|  |  |  |  |
|  |  |  |  |
